# 3D Dissection of Structural Membrane-Wall Contacts in Filamentous Moss Protonemata

**DOI:** 10.3390/ijms22010158

**Published:** 2020-12-26

**Authors:** Dominik Harant, Ingeborg Lang

**Affiliations:** Department of Functional and Evolutionary Ecology, Faculty of Life Sciences, University of Vienna, Althanstrasse 14, A-1090 Vienna, Austria; dominik.harant@hotmail.com

**Keywords:** moss, membrane-wall contact, *Physcomitrella* sp., osmotic stress, caulonema, chloronema, 3D imaging

## Abstract

In conventional light microscopy, the adjacent cell walls of filamentous moss protonemata are seen from its narrow side thereby obscuring the major area of cell–cell connection. Optical sectioning, segmentation and 3D reconstructions allow the tilting and rotation of intracellular structures thereby greatly improving our understanding of interaction between organelles, membranes and the cell wall. Often, the findings also allow for conclusions on the respective functions. The moss *Physcomitrium* (*Physcomitrella*) *patens* is a model organism for growth, development and morphogenesis. Its filamentous protonemata are ideal objects for microscopy. Here, we investigated the cell wall between two neighboring cells and the connection of membranes towards this wall after plasmolysis in 0.8 M mannitol. An m-green fluorescent protein (GFP)-HDEL cell line was used to visualize the endoplasmatic reticulum (ER), the plasma membrane (PM) was stained with FM4-64. Our studies clearly show the importance of cell–cell contacts in *P. patens* protonemata. In 86% of the investigated cell pairs, at least one of the protoplasts remained fully attached to the adjacent cell wall. By tilting of z-stacks, volume renderings and 3D reconstructions, we visualized the amount of attached/detached PM and ER components after plasmolysis and membrane piercings through the wall of cell neighbors.

## 1. Introduction

Cell-to-cell contact is essential for communication and development of multicellular organisms. A prerequisite is the passage through membranes. That way, molecular exchange and information flow is regulated via hormones, membrane proteins and pores. In plants, the rigid cell walls prevent large membrane contact areas between protoplasts. Only plasmodesmata, minute channels between adjacent cells, form direct connections. Often, molecular data of the proteins involved are manifold but there is a lack of knowledge on functional and structural information.

### 1.1. The Wall between Cells

In turgid cells, the cortical cytoplasm and the plasma membrane (PM) is pushed towards the cell wall by the turgor pressure of the central vacuole [1]. Thus, the cortical membranes are closely aligned with the cell wall and a structural or visual separation is therefore difficult. Hence, plant cell biologists use plasmolysis to induce physical detachment of the living protoplast from the cell wall [2]. Plasmolysis was first described by de Vries in 1887 [3]. Exposure of plant tissue to hyperosmotic salt or sugar solutions of about 0.8–1 M cause water efflux from the vacuole by osmosis. This water loss results in the shrinking of the protoplast and eventually the detachment of the PM from the cell wall. A reticulate network of endoplasmatic reticulum (ER) and PM remains attached to the cell wall. It is known as Hechtian reticulum [4]. Fine strands (Hechtian strands) maintain a connection of the Hechtian reticulum with the plasmolyzed protoplast [5,6]. The phenomenon of plasmolysis is frequently used in plant cell biology classes to demonstrate the PM, tonoplast and cytoplasm and the cell wall as separate structures [1,7]. Additionally, plasmolysis is a useful tool to test the viability of plant cells as only in living cells, the semipermeable membranes are intact and allow for plasmolysis. Dead cells do not plasmolyze.

Here, we used plasmolysis for the analysis of structural details at the cell wall between two neighboring cells. The focus was on the attachment/detachment of the PM (stained with FM4-64) and the ER (labeled by a green fluorescent protein (GFP)-tag). In a first step, we plasmolyzed protonema cells and defined various forms of detachment and their statistical frequency. Secondly, we looked at the two sides of the cell wall like at a door, viewed from inside the cell of neighbor one and neighbor two, respectively.

### 1.2. The Microscopic View

In the light microscope, samples are usually seen in top view. However, this microscopic view only shows the narrowest side of the walls between cell neighbors. A 90° tilt would be necessary to look at the whole disc of the wall. This is possible by confocal laser scanning microscopy (CLSM) and optical sectioning of the probe. The resulting image stacks can be tilted at arbitrary angles thereby allowing a view from inside the cell onto the wall. If labeling for specific structures has been applied, segmentation and 3D reconstructions can result in additional information, e.g., by the separation of individual structures. In our case, we imaged the labeled ER and PM in separate channels. Likewise, any other protein of interest could be imaged if it can be fluorescently marked. AMIRA^®^ was used to generate 3D reconstructions, segmentations and “helicopter views“; here, with a focus on the cell wall between two neighbors to analyze structural details of membrane-wall contact sites.

### 1.3. Moss Protonema as Model Cells

In search of the optimal samples, we chose filamentous moss protonemata [8]. These consist of a single line of cells; branching occurs only occasionally [9]. Hence, two cells normally possess only one connecting cell wall. Chloronema cells show more chloroplasts, straight cell walls and a slightly bigger diameter than caulonema cells with oblique walls [10]. In both cases, the diameter is ideal for optical stacks of the whole cell by confocal microscopy without further mechanical sectioning. Although mosses are evolutionary older than seed plants, the structural features of the cells are very similar. This also applies for plasmolysis. Additionally, in the moss model *Physcomitrium (Physcomitrella) patens*, various cell lines with GFP-tagged proteins are available (https://sites.dartmouth.edu/bezanillalab) to allow specific tracing of proteins in living samples.

In the present study, we report on the 3D reconstruction and segmentation of membranes at the adjacent wall between two neighboring cells. We give examples on the possibilities of this 3D technique to visualize structural details. Thereby, we want to inspire further studies in the field of plant cell imaging.

## 2. Results

**Figure 1:**
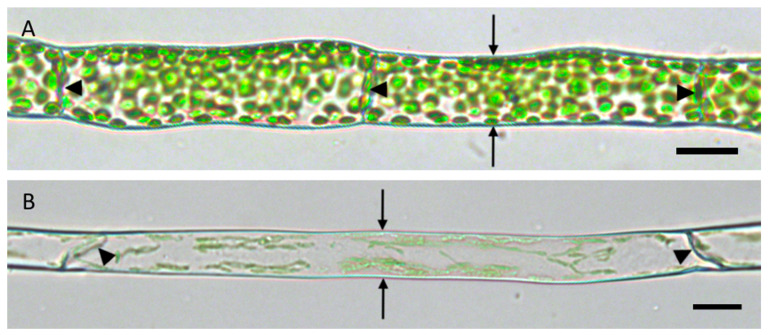
Typical shapes of *P. patens* protonema cells. (**A**) Chloronema cells with orthogonal cell wall (arrowheads) and (**B**) caulonema cells with oblique cell walls (arrowheads). The arrows point to lateral walls and show the respective diameter of the cell types. Scale bars = 20 µm.

The filamentous protonemata of *P. patens* consist of interconnected, cylindrical cells with occasional branching. Normally, two neighbors share a common middle lamella between their adjoining cell walls that is separating the individual cells. Protonemata can be distinguished into chloronema (Figure 1A) and caulonema (Figure 1B). Chloronema cells are shorter and thicker than caulonema cells. Additionally, there are differences in the abundance of chloroplasts and the inclination of the connecting cell walls. Chloronemata contain more chloroplasts and possess straight cell walls with orthogonal orientation to the growth direction. Caulonema cells are characterized by fewer chloroplasts and oblique cell walls (Figure 1).

In turgid protonemata, the PM and the cortical cytoplasm are in close alignment with the cell wall at all sides. The cortical ER consists of a polygonal network of fine tubules and sheets [11]. It has been shown to be in immediate contact with the PM [12].

Unless it is branching or a tip cell, each protonema cell has only two neighbors, one younger and one older cell, which are connected by a tilted (i.e., caulonema) or straight (i.e., chloronema) cell wall, respectively (Figure 1). It is evident that these walls are essential for information flow and exchange within the thread. Disconnection would lead to cellular isolation and a lack of transport. Here, we used plasmolysis as a tool of osmotic water loss to investigate if and how the living protoplasts detached from the cell wall. A special focus was on the essential connection between two neighbored cells and their adjoining cell wall.

**Figure 2:**
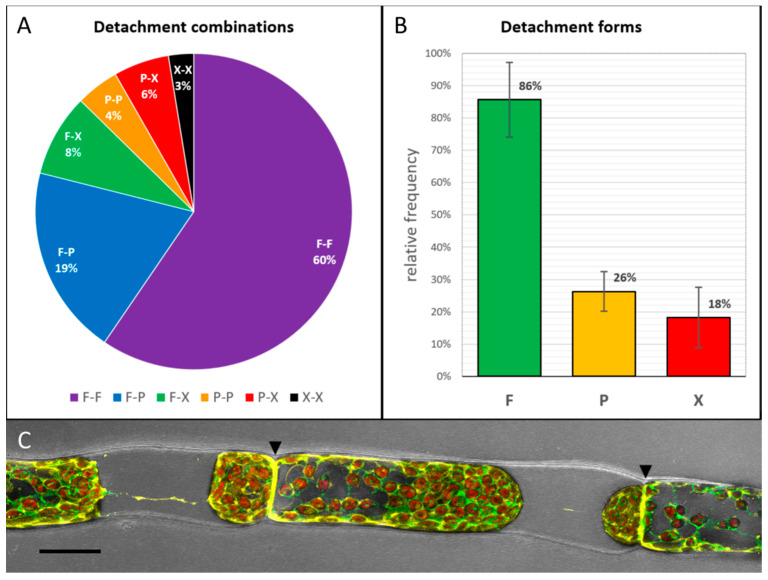
Detachment forms of protoplasts of two connected cells from their adjoining cell wall. F: fully attached, P: partially detached, X: fully detached. *n* = 603. (**A**) Detachment combinations: In 60% of the investigated cell pairs, both protoplasts remained attached at the connecting cell wall after plasmolysis. (**B**) Of plasmolyzed protoplasts 86% stayed fully attached to the adjoining cell wall, 26% remained partially attached and only 18% of cells showed full detachment. (**C**) A filamentous *P. patens* cells after plasmolysis: the protoplasts stay fully attached at both sides of the adjacent walls (arrowheads; F-F type) but are detached at the longitudinal walls. Overlay of transmission channel (grey) and fluorescence of endoplasmatic reticulum (ER; green), chloroplasts (red) and plasma membrane (PM) (yellow). Scale bar = 20 µm.

Preliminary plasmolysis experiments with 0.8 M mannitol resulted in three different detachment forms that were defined as “full attachment” (F), “partial detachment” (P) and “strong or full detachment” (X). Ergo, six different detachment combinations of protoplasts at each side of the connecting cell wall are possible: F-F, F-P, F-X, P-X and X-X. Consecutively, we examined 603 adjoining protonema cells after plasmolysis and their detachment forms (Figure 2). Both, chloronema and caulonema cells plasmolyzed without visible differences.

In 60% of the observed cell pairs, the protoplasts of both cells stayed fully attached at the adjoining cell wall (F-F type; Figure 2A,C), although the protoplasts detached from the lateral walls. These data confirm the existence of a strong membrane connection to the adjoining wall and the importance of the link between cell neighbors. The relative frequency of the three defined detachment forms showed that in 86% of the cell pairs at least one of the neighbors remained fully attached to the connecting cell wall (Figure 2B). In contrast, a strong or full detachment of at least one of the protoplasts could only be observed in 18% of the investigated cell pairs.

**Figure 3:**
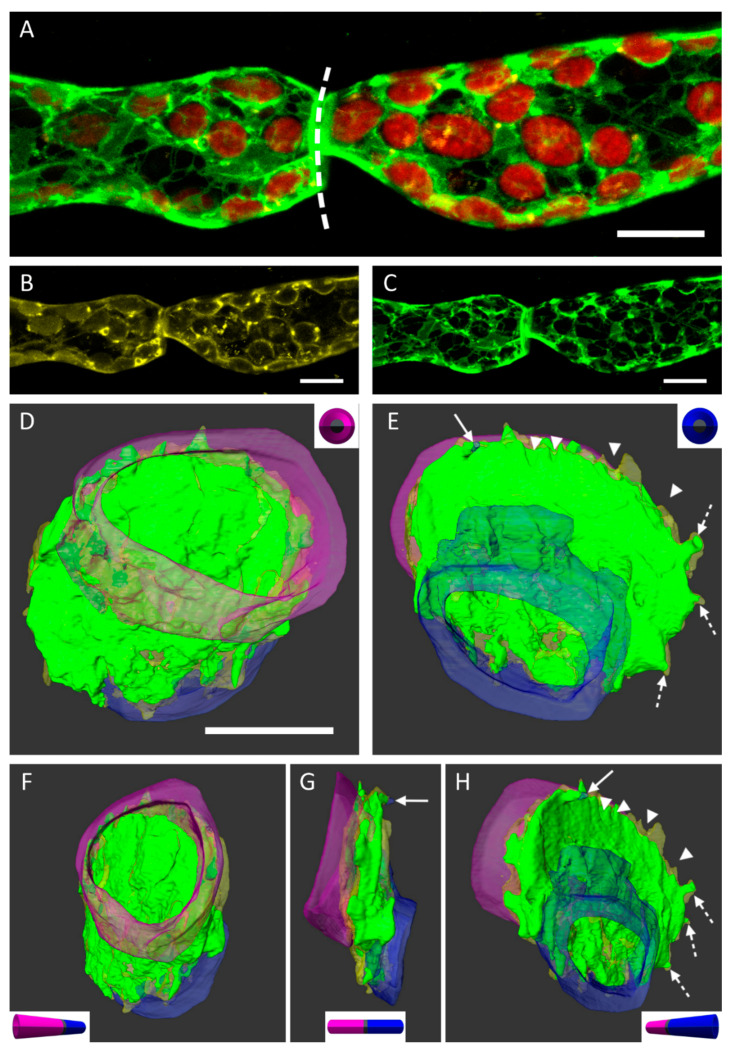
The cell wall of two plasmolyzed cells in partial detachment. (**A**) Maximum projection of a z-stack imaged by confocal laser scanning microscopy (CLSM) capturing the fluorescence of ER (green), chloroplasts (red) and PM (yellow). The dotted line indicates the approximated curvature of the cell wall between the protoplasts. (**B**) PM labeled with FM4-64. (**C**) Fluorescence of GFP-ER. (**D**–**H**) 3D reconstruction of z-stacks showing different perspectives. False-colored protoplasts in purple and blue correspond to the left and right side of the cell wall, respectively. Leftovers of the membrane and ER at the wall show a bigger connecting area of the blue protoplast and an indirect connection by another plasma strand (arrow) and via Hechtian reticula (dotted arrows). The ER and PM form little notches (arrowheads). Scale bars = 10 µm.

**Figure 4:**
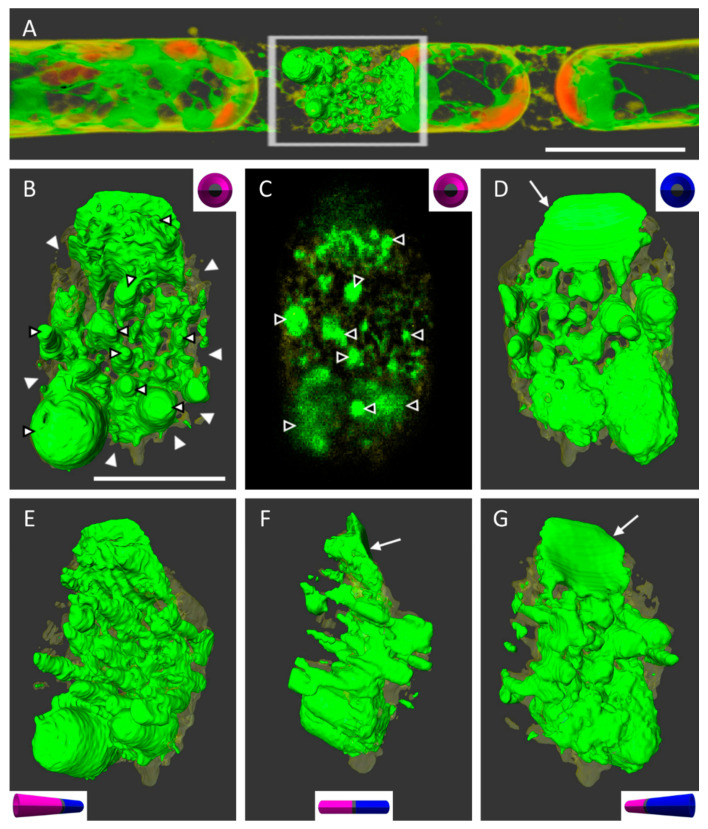
The cell wall of two adjacent caulonema cells after plasmolysis in 0.8 M mannitol. (**A**) Overview of subprotoplasts with partial 3D reconstruction of the situation at the connecting cell wall with the P-X detachment form. (**B**,**D**–**G**) Volume renderings of membranes (ER in green, PM in yellow) and views from various angles. ER and its surrounding membranes form lateral notches and little holes after plasmolyzing (white arrowheads). (**C**) A virtually generated slice through the wall, revealing a net of membrane and ER. (**B**,**C**) Some ER structures possibly extend into both cells (black-white arrowheads). (**D**,**F**,**G**) Arrows mark the protoplast attachment of the right cell (blue part in the schematic) from (**A**). Scale bar: **A** = 20 µm, **B** = 10 µm.

**Figure 5:**
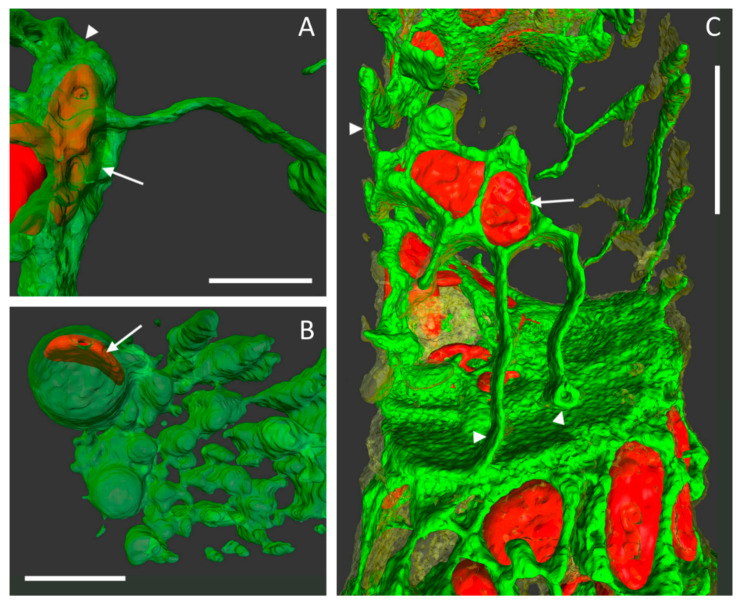
High magnification of 3D reconstructions in plasmolyzed protonema cells. ER encloses chloroplasts (arrows) as the protoplast shrinks and detaches from the cell wall. ER (green), chloroplasts (red) and PM (transparent yellow, only in **C**). (**A**) A little portion of the protoplast (arrowhead) stayed attached to the adjoining cell wall of two cells and is connected by a plasma strand with the main protoplast. Transparently colored ER encloses a chloroplast. (**B**) After increasing the transparency levels of ER stacks and bubbles, a chloroplast (arrow) is revealed. (**C**) The close up of the cell pair in Figure 3B–F reveals a partial protoplast detachment from the walls, with fine membrane strands and tubules of ER (Hechtian reticula), and even a small ring, remaining attached at the lateral cell wall (arrowheads in **C**). Scale bars: **A** and **B** = 5 µm; Scale bar: **C** = 10 µm.

Further investigations of the situation at the cell walls between two cell neighbors and particular attachments of the PM and the ER were performed by high resolution confocal imaging (Figure 3). We used a *P. patens* cell line with a GFP-ER tag (eGFP-ER; Figure 3B). The PM was stained with FM4-64, a commonly used styryl dye for cell membranes (Figure 3C). The overlay of the confocal images in 3B and 3C, including the detection of chloroplasts by the autofluorescence of chlorophyll is shown in Figure 3A. The cell wall between two adjacent protonema cells is depicted by the dashed line (Figure 3A). Optical sectioning of the sample resulted in image z-stacks, which could be implemented to generate 3D reconstructions of the observed area using AMIRA^®^ software (Figure 3D–H, Figure 4 and Figure 5). Segmentation and false-coloring the membranes gave further, more detailed insights to the three-dimensional arrangement of partially detached protoplasts. Figure 3D,E show the membrane attachments on each side of the common cell wall. Although the major part of the PM remained attached to the cell wall, the area of the two protoplasts was different at each side of the wall as seen in purple (Figure 3D) and blue (Figure 3E), respectively. Small notches (arrowheads) consisting of either ER or of PM appeared at the edges of the cell wall disc. Larger protrusions containing both membrane types were forming Hechtian strands (arrow) or were connected to the Hechtian reticulum (dotted arrows). The local shift of the two protoplasts at each side of the cell wall was only detected when comparing the situation imaged in front view (“purple” side; Figure 3F) and then in side view (Figure 3G). The number and location of emerging Hechtian strands (arrow) and Hechtian reticulum (dotted arrows) appeared by realizing tilted views from different angles (Figure 3F,H). See also supplemental Videos S1 and S2 to imaging the reconstructed cells in rotation.

In conventional light microscopy, the cross walls of cells can only be observed in the top view, i.e., by looking at the narrow side of the wall. Only after generating a 3D surface, the area of interest can be turned and observed from various angles or from an inside-the-cell perspective. Due to technical restrictions and the multitude of chloroplasts in chloronemata, the resolution in the z-direction was relatively lower than in the xy direction. To increase the resolution and image quality, we therefore switched to caulonema cells with oblique walls that laid orthogonally to the scanning direction in the xy-plane (Figure 4A). This way, any leftovers of ER and PM at the wall after plasmolysis could be analyzed more precisely (Figure 4B–G). The detailed 3D reconstructions in Figure 4 revealed the PM (in transparent yellow) surrounding the ER (shown in green) as a fine network at the adjoining wall between two cell neighbors. See also supplemental Video S3.

After plasmolysis and the detachment of the main protoplast, ER membranes remained attached to the wall by forming big bulges. Virtually generated slices through the cell wall suggested the extension of some ER structures from one side to the other thereby connecting the cell neighbors (Figure 4C).

3D reconstructions of confocal images also revealed great details of very delicate structures (Figure 5). Networks and sheet-like components of the ER and PM and Hechtian strands could be clearly distinguished. As an example, we observed several times that ER tubules and membranes closely entangled a chloroplast (Figure 5, arrows). Any Hechtian reticula and strands that remain in contact with the cell wall after plasmolysis could also be depicted in high resolution as tubules or little loops (Figure 5, arrowheads).

## 3. Discussion

In biological systems, form is intrinsically connected to function. It is therefore essential to investigate the form of specific structures in order to understand their function. Modern, computer assisted imaging tools greatly support the visualization of structural details at all levels of complexity [13,14]. 3D presentations of biological structures inspire the imagination of the audience and are a fantastic asset, not only to solve scientific questions but also for teaching [2].

In the present study, we focused on the structure of PM and ER membranes at the adjacent cell wall of two connected protonema cells. In interphase cells of *P. patens* protonemata, the PM is usually closely aligned with the cell wall and the cortical ER forms are a dynamic network of tubules and sheets [2,15], as is also the case in cells of seed plants. A membrane-wall continuum between plant cell neighbors is formed by plasmodesmata [16]. Additionally, various proteins like reticulons [17,18,19], reticulon-like proteins and atlastins [20] can shape the ER. Others provide ER–PM membrane contact sites [6] and connect the ER and PM to the cell wall, also to the parts of the cell wall that do not possess adjacent cells. The nature of these linkers is not fully understood, but they could be the anchor points for Hechtian strands and reticulum at the cell wall. Additionally, Lang et al. proposed a physical anchor by cellulose fibers to link the plasma membrane to the cell wall [5] and also the cytoskeleton is involved [2,6]. In all these cases, the visualization of the situation remained challenging in turgescent cells and therefore, we used plasmolysis for physical separation of the protoplast from the cell wall. This way, we could image the structural differences of cortical membranes and the cell wall. Additionally, segmentation of the z-stacks, image rotation and 3D reconstructions greatly help to distinguish structural differences. Particularly in the case of the adjacent walls in protonema cells, the connecting wall is normally observed from its narrow side as a line (Figure 1) and not as a disc as is possible after 90° rotation of optical sections. To visualize the obscured parts, it is essential that the resolution in z direction is minimal and at least the same as in the xy direction in order to prevent image distortions.

Our data clearly show that cell–cell contact is essential in filamentous cells (Figure 2). In 86% of the investigated cases, at least one of the neighbors remained fully attached to the cell wall after plasmolysis (Figure 2B). A strong connection of the protoplast to one side of a cell has been described as “negativer Plasmolyseort” (location of negative plasmolysis; [21,22]). In *Avena sativa* coleoptiles, the amount of plasmodesmata correlated positively with the adhesion of the plasmolyzed protoplast [7]. Plasmodesmata have been proposed to provide connection sites for Hechtian strands and the Hechtian reticulum [23]. Bryophytes also possess plasmodesmata with desmotubules [24] and a membranous connection between cell neighbors in *P. patens* is also confirmed here. Virtually generated slices of the cell wall in caulonema cells clearly show transversing membranes between the adjacent cells (Figure 4). However, the reasons for the strong membrane–wall connection at the walls between cell neighbors in *P. patens* protonemata are still not fully understood and it remains unclear why the contact is sometimes lost and how the disconnected/connected areas are defined.

As depicted in Figure 3, segmentations of the protoplasts show differences in the connecting areas at each side of the cell wall; the purple protoplast was larger than the blue protoplast (Figure 3E). There was a narrow part of the blue protoplast that maintained the contact in the center of the cell wall disc (Figure 3F). Additionally, image tilts and reconstructions of the cell wall in Figure 3 revealed strands of PM and ER and small notches at the edge of the cell wall disc. These were visible at the corner of protonema cells, distant from the plasmolyzed protoplasts. It remained to be tested if these structures colocalize with cytoskeletal elements like specific microtubules that bend at the cell corners [25] or leftovers of zones that evolved from phragmoplast formation [26].

High resolution z-stacks allow for 3D imaging of fine, structural details and the positioning of organelles within membranes (Figure 5). Volume renderings in combination with varying transparency levels reveal that the chloroplasts are closely entangled by networks and sheets of ER membranes [27], and that the membranes of ER and PM are distinct and separate from each other.

The images of the present study should serve as an example: depending on the scientific question, other proteins or structures of interest could be labeled and then analyzed as described here. Furthermore, segmentation and 3D imaging is not restricted to CLSM z-stacks. Segmentation software like AMIRA^®^ usually accept also data sets originating from other research fields or imaging techniques like micro computed tomography (µCT) [28] transmission electron microscopy (TEM; [29] or focused ion beam scanning electron microscopy (FIB-SEM), as shown for the green alga *Micrasterias detenticulata* [13] or *Klebsormidium* sp. [14].

## 4. Material and Methods

### 4.1. Moss Culture and Sample Preparation

The moss *Physcomitrium patens* (Funariaceae) was cultivated in sterile tissue cultures in a PpNH_4_-moss medium, containing MgSO_4_·7H_2_O, KH_2_PO_4_, CaNO_3_·4H_2_O, FeSO_4_·7H_2_O, Di-Ammoniumtartrate and microelements [30]; all chemicals were obtained from Sigma-Aldrich, Austria. Every five weeks, we inoculated small protonema pieces into new agar plates. The resulting subcultures were preserved in a growth cabinet at 20 °C with 50% relative humidity and a 14 h light/10 h dark cycle. For our investigations, some parts of the protonemata were placed on a glass slide between two Vaseline^®^-stripes. The samples were covered by a glass coverslip; the Vaseline^®^ preventing the cells to be squashed between the glasses. Consequently, two open ends were created, allowing a facile application of the plasmolytic solution. The respective chloronema or caulonema cells for the experiments were selected directly from the slides.

### 4.2. Fluorescence Markers and Staining

We analyzed the behavior of the plasma membrane (PM), the endoplasmatic reticulum (ER) and the chloroplasts in plasmolysis. We used protonemata of a *P. patens* cell line, which expresses mGFP:ER:HDEL, kindly provided by Magdalena Bezanilla [30]. This line is expressing mEGFP targeted to the ER-protein HDEL. The autofluorescence of chlorophyll allowed the simultaneous observation of chloroplasts without additional staining. For the visualization of the PM and Hechtian structures, we stained the protonema cells with 36 µM FM4-64 (ThermoFisher Scientific, Waltham, MA, USA) for 60 min [2]. We washed the cells 3–4 times with 100 µL distilled water prior to direct observation or transfer to a hyperosmotic solution.

### 4.3. Plasmolysis

Hyperosmotic treatment with a 0.8 M mannitol solution resulted in plasmolysis of *P. patens* protonema cells. After staining as described above, we applied 100–150 µL of the plasmolysis solution and soaked it through the Vaseline^®^ channel directly on the slide to ensure mannitol exposure around the protonema cells. To antagonize variations of the solution’s osmolarity, the open ends of the Vaseline^®^ chamber were always covered by a mannitol film [2]. Microscopy of the ER, the chloroplasts and the PM took place after at least 30 min of mannitol treatment.

### 4.4. Confocal Laser Scanning Microscopy (CLSM)

Live cell imaging was performed with an upright confocal laser scanning microscope (Leica TCS SP5 DM-6000 CS, Leica Microsystems, Vienna, Austria) and the connected LAS AF Software v4 (Leica Microsystems, Vienna, Austria). All images were taken with a 63× water immersion objective (NA 1.2). We used a multi-argon laser and selected a wavelength of 488 nm for excitation of the different fluorochromes. The emission wavelengths of GFP-ER (495–550 nm), FM4-64 (575–640 nm) and chlorophyll (670–770 nm) were detected by three different multipliers simultaneously. The focal depth was set to about 0.5 µm and the pinhole was adjusted to one airy disc. To obtain high resolution images without motion blurring, a scanning speed of 200 or 400 Hz were chosen. Single images were edited with FIJI software [31]. Optical sectioning in the z-direction was performed in the same resolution as in the x–y direction and resulted in z-stacks of 100–300 images, which were consecutively used to generate 3D reconstructions of the detected fluorescent cell structures.

### 4.5. 3D-Reconstruction

High-resolution 3D-models of the ER, PM and chloroplasts of plasmolyzed chloronema and caulonema cells, were obtained by setting the step size in z-direction to “system optimized” (0.13 µm) in order to match with the resolution in the x–y direction. Z-stacks were then transferred to the 3D visualization program AMIRA^®^ 6.2.0 (FEI, Hillsboro, Oregon, USA). Subsequently, the detected cell structures were segmented with the threshold tool to generate a pseudo-colored and smoothed surface. This way, we could analyze the labeled membranes and chloroplasts even further. To highlight the attachments of the protoplasts, we marked the membrane in this area by hand using the brush tool. At the adjoining cell wall, the ER is shown in green and the plasma membrane as transparent yellow with its surface generated by a threshold-tool. Twenty to thirty cell pairs were segmented and reconstructed in this way.

**Figure 6:**
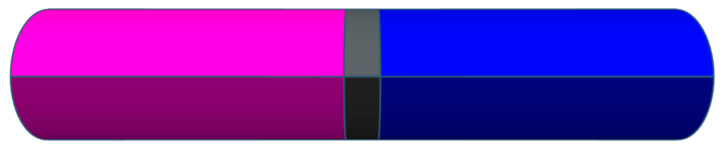
Cell model to illustrates two neighbors of filamentous protonema cells (purple and blue, respectively) and their connecting cell wall (grey). Z-scans always started from the bright, upper side.

For better visualization and orientation particularly in the 3D images showing tilted cell walls, we added a cylindric 3D model of two adjacent cells (Figure 6). The left cell is in purple, the right cell in blue, and the adjacent cell wall in grey. The model was created in the online application TinkerCAD (Autodesk Inc., San Rafael, CA, USA).

### 4.6. Detachment Forms and Statistics

Quantitative analyses of the detachment forms of protoplasts from their adjoining cell walls after plasmolysis were performed in 603 neighboring cell-pairs of chloronemata from 15 different samples. Plasmolysis times were between 60 and 100 min of mannitol treatment. Furthermore, only the youngest 10 cells per protonema filament were taken into account, given that they have plasmolyzed. Protonema tip cells and branching cells were excluded. The nominal-scaled data were analyzed and visualized in Microsoft Excel 2019 (Microsoft Cooperation). To detect data outliers, we excluded values beyond 1.5× of the interquartile range (IQR). 

## 5. Conclusions

The possibilities and the quality of 3D imaging of CLSM data has changed massively since the early 1990s. Confocal imaging is still an unsurpassed tool for 3D visualization when working with living cells. The 3D-models obtained greatly help to image and analyze certain subcellular structures. The technique, however, is not restricted to the examples of PM and ER in plasmolyzed *P. patens* protonemata.

However, we report a very strong, physical contact between the two adjacent protonema cells in 60% of the cases. Not only the PM but also the ER play a major role in the form and quality of this attachment to the cell wall.

## Data Availability

Data are available upon request.

## Supplementary Materials

The following videos are available online at https://www.mdpi.com/1422-0067/22/1/158/s1, Video S1: 3D reconstruction of two adjacent *P. patens* chloronema cells after plasmolysis (corresponding to Figure 3B–F). The viewer is virtually travelling through the sample in the yz-direction. The segmented 3D model shows the partial detachments (P–P) at the adjoining cell wall. Video S2: Rotating 3D reconstruction of the membrane attachments at the cell wall between two chloronema cells (corresponding to Figure 3B–F). Video S3: 3D reconstruction of two neighbored *P. patens* caulonema cells after plasmolysis (corresponding to Figure 4). In a virtually generated movie, the sample is observed in the yz-direction. We appreciate the high resolution 3D model of a fine ER and membrane network at the wall of two adjacent cells.
